# The impact of body position on vital capacity among pregnant women in the second trimester

**DOI:** 10.3389/fmed.2024.1351681

**Published:** 2024-05-27

**Authors:** Raid Al Zhranei, Shatha Alsulami, Weam Alfaydi, Reem Alzahrani, Maher Alsulami, Ziyad F. Al Nufaiei

**Affiliations:** ^1^Respiratory Therapy Department, College of Applied Medical Sciences-Jeddah, King Saud bin Abdulaziz University for Health Sciences, Jeddah, Saudi Arabia; ^2^King Abdullah International Medical Research Center (KAIMRC), Riyadh, Saudi Arabia; ^3^Emergency Medical Service, College of Applied Medical Sciences-Jeddah, King Saud bin Abdulaziz University for Health Sciences, Jeddah, Saudi Arabia

**Keywords:** slow vital capacity, the second trimester, pregnancy, spirometry, body mass index

## Abstract

**Background:**

Pregnancy introduces significant physiological changes, notably impacting respiratory dynamics, especially during the second trimester. Data remain inconclusive about how body posture might influence lung function in pregnant women. We aimed to examine the impact of body position on slow vital capacity in pregnant women during their second trimester.

**Methods:**

This observational study was carried out at King Khalid Hospital in Saudi Arabia, involving pregnant women in their second trimester, from 14 to 26 weeks of gestation. We utilized the KoKo® Legend Portable Office Spirometer to measure slow vital capacity (SVC) in both sitting and standing positions. Participants’ demographic details were recorded, ensuring a comprehensive analysis that accounted for age, BMI, and gestational age.

**Results:**

136 pregnant women participated in this study, a paired-sample *t*-test revealed no statistically significant difference between sitting (M = 2.31, SD = 0.49) and standing (M = 2.33, SD = 0.5) positions, *p* = 0.24, However; the mean value of SVC in sitting position was significantly different between 4th month of pregnancy (M = 2.17, SD = 0.44) and 6th month of pregnancy (M = 2.45, SD = 0.48), *p* = 0.016.

**Conclusion:**

The performance of the SVC in both positions was not significantly affected. However, an increase in gestational age had a notable impact on SVC performance, particularly during sitting positions, due to the changes in respiratory physiology during pregnancy.

## 1 Introduction

Pregnancy fundamentally transforms a woman’s life, bringing about a multitude of physiological and anatomical changes necessary for the developing fetus. Respiratory mechanics experience significant changes as metabolic demands increase and design evolves, representing some of the most notable alterations (1, 2). The second trimester, occurring between weeks 13 and 26, represents a pivotal period where these physiological adaptations experience notable changes (1, 2). Vital capacity is an essential factor in respiratory function as it assesses the maximum amount of air that can be exhaled after taking a deep breath (3). Various factors, including body posture, can potentially impact vital capacity, a key indicator of lung health (3). It is important for pregnant women to understand how different body positions affect their vital capacity. This can help them improve their respiratory well-being during pregnancy (3, 4).

A study by Patel et al. (5) investigated the impact of different body postures on respiratory function during pregnancy, focusing specifically on dyspnea, a common issue caused by physiological changes. The researchers evaluated Peak Expiratory Flow Rate (PEFR), a key measure of lung function, among 72 participants between 14 and 27 weeks of gestation while in various relaxation postures. The findings revealed that relaxed standing, sitting, or high side-lying positions were associated with decreased PEFR levels, whereas front-lean standing, or forward lean sitting positions resulted in the highest PEFR values. Notably, upright postures consistently yielded higher PEFR results compared to recumbent positions, suggesting that adopting specific body positions may benefit respiratory health during pregnancy (5). Evidence suggests that expecting mothers can alleviate respiratory difficulties by adopting a forward-leaning posture while standing, sitting, or lying down. This significant finding offers crucial guidance for combating dyspnea and improving overall comfort for pregnant women (5–7).

Another study by Harirah et al. (8), examined the effects of gestational age and maternal position on peak expiratory flow rates (PEFR) in pregnant women. By recording PEFR values in standing, sitting, and reclined positions throughout pregnancy and after delivery, the researchers observed a substantial decline in PEFR with increasing gestational age (8). This decline was more pronounced in the supine position compared to standing or sitting postures. Notably, PEFR levels reached approximately 71.9% of early pregnancy values after childbirth. The study underscores the importance of considering maternal position and gestational age when interpreting PEFR measurements, particularly in pregnant women with asthma (8).

During pregnancy, the organs’ organization and function undergo significant changes. During the first trimester of pregnancy, the uterus is located in the pelvic cavity. However, as the pregnancy develops during the second and third trimesters, the uterus moves into the belly, significantly altering the size of the pelvic and abdominal cavities (9). The abdominal organs grow and reorganize, lengthening the diaphragm, a vital muscle for breathing. When stretched, the diaphragm thins in the opposite direction to the tension. The Poisson’s ratio notion is appropriate in this situation. The expanding uterus’s evenly rising pressure on the diaphragm’s abdominal surface causes it to lengthen when in posture (10, 11).

The rising abdominal pressure also suggests an increased inspiratory load on the diaphragm during pregnancy. The expanded diaphragm and the greater load indicate that the muscle contracts eccentrically during inhaling (3, 4). The diaphragm may ultimately get stronger and more conditioned because of these eccentric contractions that take place during normal breathing; this is vital during childbirth, when the diaphragm is crucial (4, 9). It is crucial to assess respiratory function in pregnant women, especially during the second trimester. We aimed to bridge the gap in knowledge by conducting thorough research on the impact of body position on slow vital capacity (SVC) in pregnant women during their second trimester.

## 2 Methods

### 2.1 Study design and setting

This study was conducted at King Khalid Hospital in Jeddah City in Saudi Arabia that is located at zero-level elevation. A comparative cross-sectional study design was employed to evaluate the impact of body position on vital capacity among pregnant women in their second trimester.

### 2.2 Recruitment of the study population

All pregnant women who visited the maternity care and family clinic at King Khalid Hospital between March 2022 and February 2023 and met our eligibility criteria were invited to participate in this study. Our eligibility criteria included: (1) a confirmed pregnancy in the second trimester, (2) No history of pulmonary diseases such as asthma, Chronic Obstructive Pulmonary Disease (COPD), (3) No history of smoking, and (4) No sign of lordosis, kyphosis, and scoliosis. To ensure eligibility, they were asked to complete a demographics form with specific information on their age, height, weight, ethnicity, number of months into pregnancy, pregnancy history, and medical history.

The participants were provided with comprehensive information about the study and potential side effects, including temporary feelings of dizziness, lightheadedness, trembling, and fatigue. Each participant signed the consent form before the procedure. The procedure was thoroughly explained to each participant twice. During the initial session, the procedure was briefly explained, providing a clear and concise overview. In the following session, a practical demonstration was conducted, right in front of the participant.

The participants were given specific instructions to maintain the correct posture while performing both the sitting position test and the standing position test. To ensure accurate calculations, it is necessary to conduct a minimum of three trials for each position.

### 2.3 Sampling

G-power software was used to determine the effect size, considering the mean and standard deviation from a pilot study (12). Based on that, the effect size was 0.24, the level of significance (*p*-value) of probability < 0.05, and the power set to 0.8. According to Cohen’s guidelines, the effect size is considered small. However, a small effect is smaller than a medium but not too small to be insignificant. The estimated sample size was 109 participants.

### 2.4 Instruments

The KoKo® Legend Portable Office Spirometer was used for measuring SVC, adhering to the most recent ATS/ERS spirometry guidelines (13). Participants were guided by both the ATS/ERS technical standards and supplementary instructions from the American Lung Association to enhance their understanding of the spirometry test procedures and comfort with the test procedure, focusing on ensuring a calm and consistent breathing pattern before and during the test (14). This dual-guideline approach was chosen to optimize test accuracy and participant experience (13, 14). To conduct the SVC tests, participants underwent a brief resting period to ensure baseline respiratory status. They were then instructed to take a deep breath and exhale into the spirometer’s mouthpiece as completely as possible, following a specific sequence designed to maximize lung capacity measurement accuracy (13, 14). A minimum of three maneuvers should be obtained to ensure consistency and reliability in the measurements obtained. Technically, the three trials should be within 0.150 L. Then, the report should include the highest value obtained from a minimum of three acceptable maneuvers (13, 14). The NHANES spirometry protocol was also used, which measures lung function using standard spirometry measurements. Predicted values were calculated based on age, height, gender, and ethnicity.

### 2.5 Operational definitions

#### 2.5.1 Second trimester

It is determined by gestational age that lasts from weeks 13 to 26 of pregnancy (1, 2).

#### 2.5.2 Slow vital capacity

The volume of air that can slowly be exhaled after maximum inspiration (3, 4, 14).

#### 2.5.3 Body mass index

It is a special measurement utilized by health care providers to determine categories of obesity for the patient based on an individual’s height and weight (15). According to the World Health Organization (WHO), the BMI is classified as follows: less than 18.5 kg/m^2^ is underweight; 18.5–24.9 kg/m^2^ is normal weight; greater than or equal to 25.0 kg/m^2^ is overweight, and greater than or equal 30 kg/m^2^ is obesity (15).

### 2.6 Statistical analysis

Data were analyzed using SPSS version 28 (IBM Corp., Armonk, NY, United States). Descriptive statistics, including frequencies, and percentages were calculated to summarize the demographic characteristics of the participants, such as age, BMI, gestational age, and parity status. Prior to statistical testing, the normality of the data distribution for SVC values was assessed using the Kolmogorov–Smirnov test (16). As the data followed a normal distribution, parametric tests were employed for further analysis.

The primary analysis involved comparing the mean SVC values obtained in the sitting and standing positions for each participant. To account for the repeated measures design, where each participant underwent both posture conditions, the paired-samples *t*-test was used. This statistical test allowed for the evaluation of any significant differences in SVC between the sitting and standing positions while accounting for the within-subject variability (17). Secondly, with the aim of assessing the relationship between BMI and SVC in both positions, the Pearson product–moment correlation coefficient was used. Finally, one way ANOVA was used to compared difference between months of pregnancy and both positions. All statistical tests were two-tailed, and a *p*-value of less than 0.05 was considered statistically significant.

### 2.7 Ethical consideration

The study (SP22J/128/08) was approved by the IRB committee of King Saud bin Abdulaziz University for Health Sciences. Each participant performed the test privately; no personal data were obtained. The participants were informed that their involvement in the study could potentially lead to the publication of a paper, and they were explicitly reassured of their right to withdraw at any given time. In this study, guidelines outlined in the Declaration of Helsinki were followed. The research team underwent comprehensive training to carry out spirometry tests with precision. In addition, stringent safety measures were implemented to address and mitigate any potential complications associated with the procedure.

## 3 Results

### 3.1 Demographic characteristics

The study sample consisted of 136 pregnant women in their second trimester. The majority of participants (61.8%) were between 22 and 29 years of age, with a smaller proportion (6.6%) above 40 years of age. Regarding BMI, 29.4% of the participants were fall between 18.5 and 24.9 kg/m^2^, as normal weight. However, 66.1% of the participants were above the normal weight range of 25 kg/m^2^. In terms of gestational age, most participants (39.7%) were in their sixth month of pregnancy, followed by 33.1% in their fourth month and 27.2% in their fifth month. Notably, 41.2% of the participants were experiencing their first pregnancy, while the remaining participants 58.8% had previous pregnancies (Table 1).

**Table 1:** Demographic characteristics of participants.

	No. of participants (*n* = 136)	%
Age Group		
22–29	84	61.8
30–39	43	31.6
40–49	9	6.6
BMI		
<18.5	6	4.4
18.5–24.9	40	29.4
25.0–29.9	51	37.5
≥30	39	28.6
Month of pregnancy		
4th	45	33.1
5th	37	27.2
6th	54	39.7
Number of pregnancies		
1	56	41.2
2	39	28.7
3	17	12.5
4	10	7.4
5	10	7.4
7	4	2.9

### 3.2 The assumption of normality

**Figure 1:**
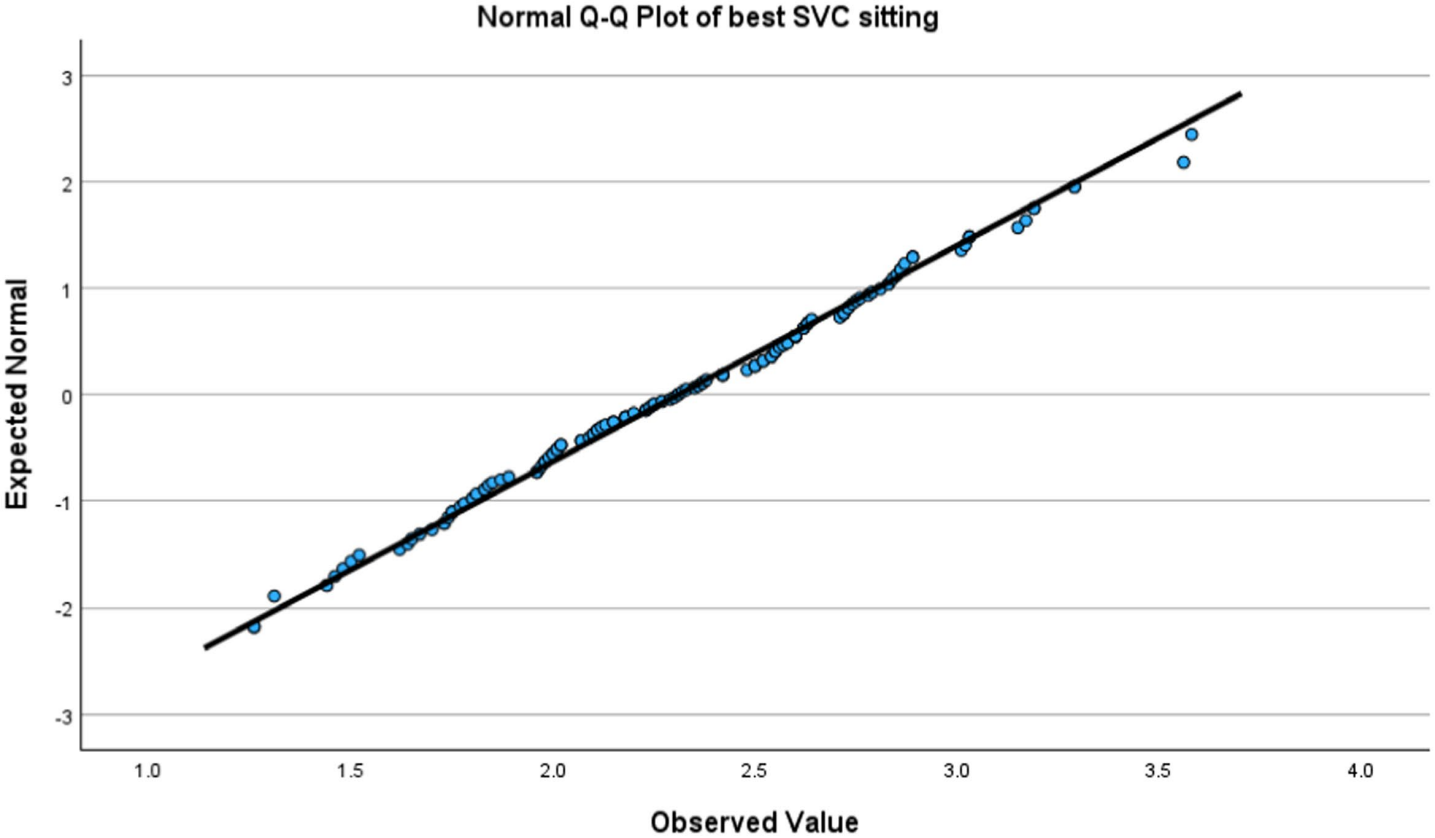
Normal Q-Q Plot of SVC at sitting position.

**Figure 2:**
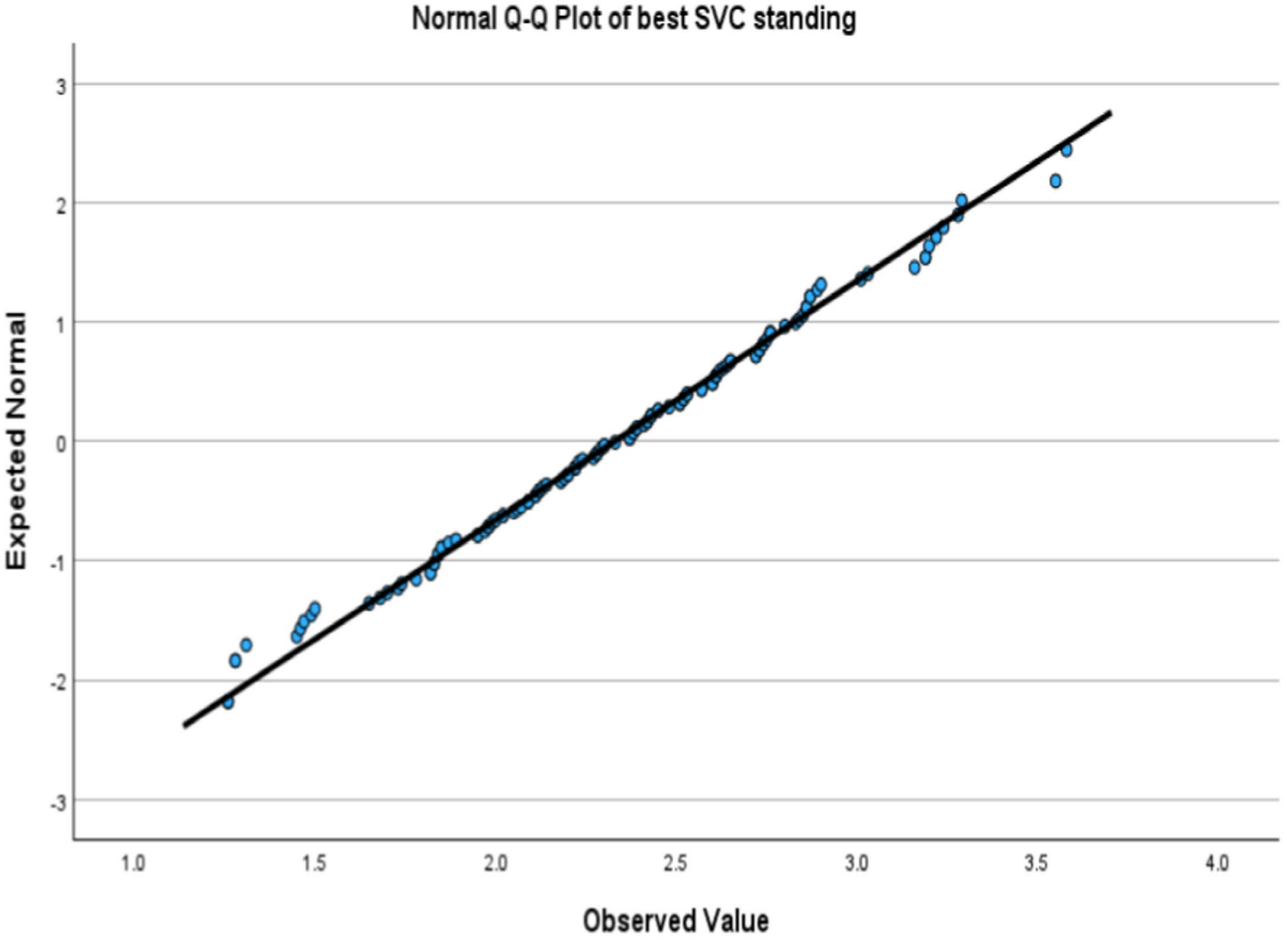
Normal Q-Q Plot of SVC at standing position.

Kolmogorov–Smirnov test showed non-significant which indicated a normal distribution. Figures 1, 2 showed a lovely straight diagonal line that indicated the actual value as same as the expected value; then the variable was normally distributed in both positions.

### 3.3 Comparison of the participant’s performance in both positions

A total of 136 participants performed three trials of SVC measurements in both the sitting and standing positions. The participants’ performance during the sitting position had an average SVC of 2.31 L, slightly less than their average performance during the standing position, which was 2.33 L. However, a paired-sample t-test revealed no statistically significant difference between the mean SVC values in the sitting (M = 2.31, SD = 0.49) and standing (M = 2.33, SD = 0.5) positions, *t*(135) = −1.19, *p* = 0.24 as shown in Table 2. The mean SVC value in the sitting (M = 2.31) and standing positions (M = 2.33) are relatively close, with a slight difference of 0.02 L favoring the standing position.

**Table 2:** Paired-sample test.

					95% confidence interval of difference		
Measurement	Positions	Mean value ± St. Dev	*t*	df	Lower	Upper	*p* value
SVC (L)	Sitting	2.31 ± 0.49	−1.186	135	−0.047	0.012	**0.238**
	Standing	2.33 ± 0.5					

Bold values indicate that if the value is lower than 0.05, there’s a significant difference, if it is above 0.05, there is no significant difference.

### 3.4 Relationship between BMI and SVC in both positions

The Pearson correlation coefficient was used to assess the relationship between BMI and SVC in both positions. As a result displayed in Table 3, the BMI was not significantly correlated with SVC in a sitting position, *r* = 0.078, and standing position, *r* = 0.095, (all *ps* > 0.005).

**Table 3:** Correlations.

		SVC sitting	SVC standing	BMI
BMI	Pearson correlation	0.078	0.095	1
	Sig. (two-tailed)	0.364	0.271	
	*N*	136	136	136

### 3.5 Difference among months of pregnancy

**Figure 3:**
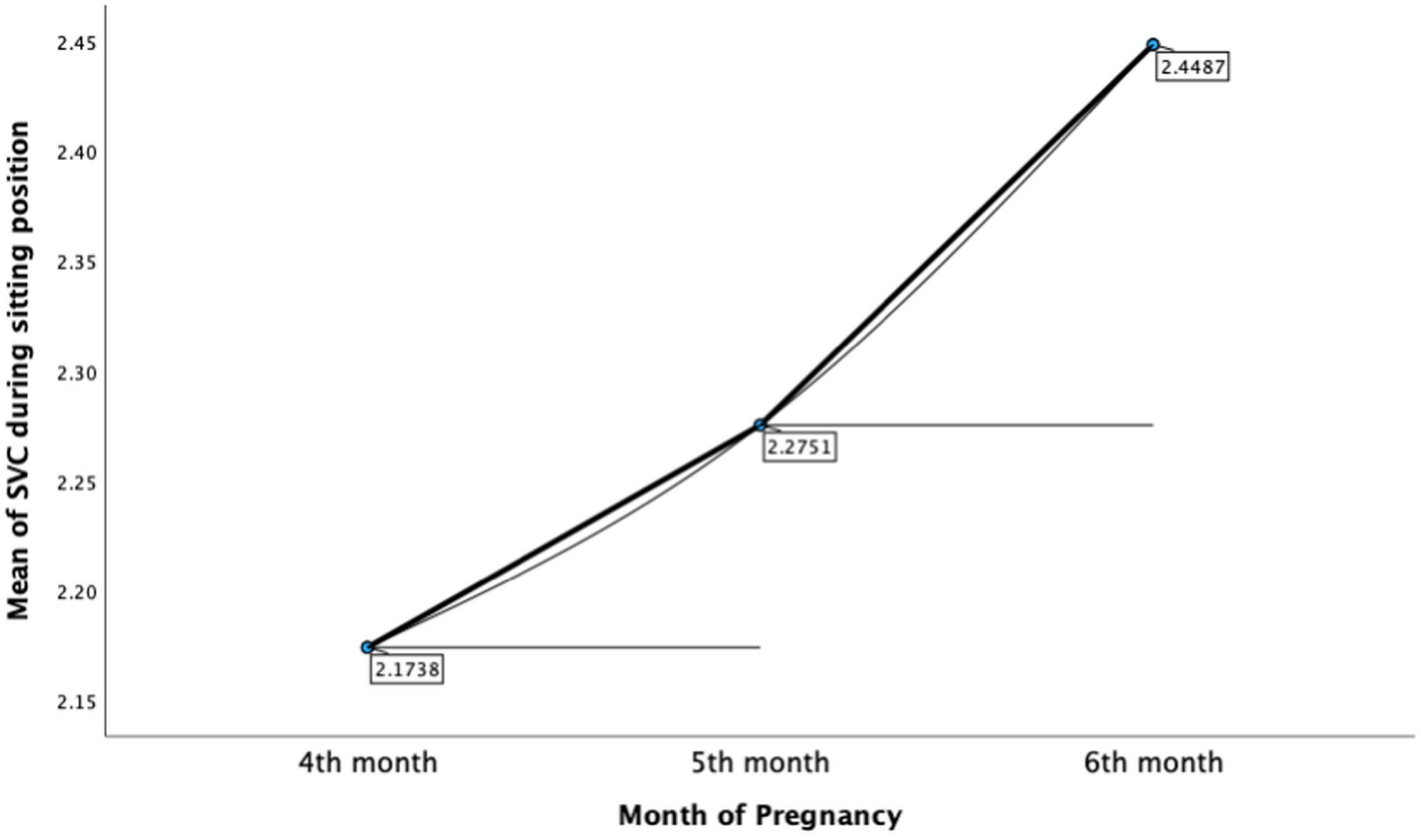
Means plots of SVC during sitting position.

The ANOVA test was performed to assess the difference between months of pregnancy (4, 5, and 6th month) with SVC in both positions. As displayed in Table 4, there was a significant difference between months of pregnancy and participants’ performance of SVC in sitting position, *F*(2,133) = 4.135, *p* < 0.05. A pairwise comparison was applied to compare different among three groups (4, 5, and 6th month; Table 5). Bonferroni’s test for multiple comparisons found that the mean value of SVC in the sitting position was significantly different between 4th month of pregnancy (M = 2.17, SD = 0.44) and 6th month of pregnancy (M = 2.45, SD = 0.48), *p* = 0.016, 95% C.I. = [−0.51, −0.04] (Figure 3). For the standing position, there was no significant difference between months of pregnancy and participants’ performance of SVC in standing position, *F*(2,133) = 2.909, (*p* = 0.06) (Table 6).

**Table 4:** ANOVA.

SVC sitting					
	Sum of squares	df	Mean square	*F*	Sig.
Between groups	1.919	2	0.959	4.135	**0.018**
Within groups	30.860	133	0.232		
Total	32.779	135			

Bold values indicate that if the value is lower than 0.05, there’s a significant difference, if it is above 0.05, there is no significant difference.

**Table 5:** Multiple comparisons.

Dependent variable: SVC sitting							
	(I) Month of pregnancy	(J) Month of pregnancy	Mean difference (I-J)	Std. error	Sig.	95% Confidence interval	
						Lower bound	Upper bound
Bonferroni	4th month	5th month	−0.10136	0.10690	1.000	−0.3605	0.1578
		6th month	−0.27493^*^	0.09723	**0.016**	−0.5107	−0.0392
	5th month	4th month	0.10136	0.10690	1.000	−0.1578	0.3605
		6th month	−0.17357	0.10280	0.281	−0.4228	0.0757
	6th month	4th month	0.27493^*^	0.09723	**0.016**	0.0392	0.5107
		5th month	0.17357	0.10280	0.281	−0.0757	0.4228

^*^The mean difference is significant at the 0.05 level. Bold values indicate that if the value is lower than 0.05, there’s a significant difference, if it is above 0.05, there is no significant difference.

**Table 6:** ANOVA.

SVC sitting					
	Sum of squares	df	Mean square	*F*	Sig.
Between groups	1.411	2	0.706	2.909	0.06
Within groups	32.267	133	0.243		
Total	33.678	135			

## 4 Discussion

Our study examined 136 s-trimester pregnant women, to evaluate impact of sitting and standing positions on SVC. The majority of the participants were in their sixth month of pregnancy, with a mix of first-time and experienced mothers. Despite this diverse demographic, our analysis of SVC measurements in different postures—sitting versus standing—revealed no statistically significant differences. This suggests that body posture may not significantly impact lung function in this specific group of pregnant women. Our findings diverge from previous research by Patel et al. (5), which suggested that posture might influence breathing efficiency in pregnancy, with certain positions possibly aiding in alleviating respiratory discomfort. Our study’s contrasting outcome could be attributed to the different gestational stages of the participants. The majority of our participants were in the later stages of the second trimester, suggesting that the effect of gestational age on lung capacity might be more pronounced as pregnancy progresses.

Additionally, this study had a large group of participants who were not primigravida (58.8%), which significantly affects the results as primigravida are experiencing the pregnancy trimesters for the first time, having a heightened experience for every change in their body in comparison to non-primigravida participants who have already experienced pregnancy and are aware of the body changes to expect, thus, may have adapted accordingly. Another study conducted by Ruhighara et al. (18), which aimed to analyze the spirometry profiles among pregnant and non-pregnant African women reported that spirometry test values are lower in pregnant than in non-pregnant participants. However, the study also reported that the spirometry profile was higher in parous than in nulliparous women which aligns with our study findings (18).

The BMI is an essential variable that may impact the performance of the SVC during the pregnancy. However, our study did not yield any significant association between BMI and SVC in both positions. This indicates that BMI may not have a substantial impact on lung function in pregnant women during the second trimester. Our findings are consistent with previous studies that also supported this conclusion (19, 20). Additionally, the study also examined the variation in SVC among pregnant women at various gestational ages, with a particular emphasis on the fourth, fifth and sixth months of pregnancy. Based on our study, there was a significant difference in SVC between the fourth and sixth months of pregnancy in the sitting position. The sixth-month individuals had higher SVC values. On the other hand, no appreciable variations in SVC were noted in the standing position over the several pregnant months. Because of the cumulative physiological changes that occur as pregnancy progresses (4, 7, 19–21), our findings suggest that gestational age may have a more apparent effect on lung function in specific body positions.

While this study offers valuable insights, it is essential to acknowledge its limitations. The relatively small sample size of 136 participants in this study may limit the generalizability of the findings to the broader population of pregnant women. In addition, the exclusion of women with lung diseases or a history of smoking limits the application of findings, even if it is necessary. Further studies of such nature should examine these effects in a more diverse population. Moreover, potential confounding factors such as physical activity levels and pre-existing medical conditions. The impact of lifestyle factors, especially physical exercise, on respiratory function has been reported in the literature. For example, Leite et al. (22) found that physical activity modulates cardiovascular and metabolic responses during pregnancy, implying that it may also have an indirect impact on respiratory dynamics. Similarly, pre-existing medical disorders, even if not directly related to pulmonary function, may alter physiological adaptations during pregnancy (21). Furthermore, longitudinal studies tracking respiration changes in each trimester would provide a complete understanding and would be further elaborated by comparing multiparous women to primigravida women (4, 11). This research can help fine-tune advice for physicians and the pulmonary function technologists to conduct pulmonary function tests for pregnant women, especially in late second trimester, in a standing position.

## 5 Conclusion

Our study contributes significant insights into how body position affects a woman’s SVC during the second trimester of pregnancy. While a combination of primigravida and multiparous women were among our subjects, there were no statistically significant differences in SVC between sitting and standing positions. Furthermore, neither the standing nor the sitting positions showed a statistically significant correlation with BMI and SVC, indicating that BMI may not have a substantial impact on lung function in the second trimester of pregnancy. Conversely, the progression of SVC underwent significant changes from the fourth to sixth months of pregnancy when comparing the sitting position with the standing position. These changes can be attributed to the cumulative physiological transformation that occur as pregnancy advances.

## Data availability statement

The raw data supporting the conclusions of this article will be made available by the authors, without undue reservation.

## Ethics statement

The study (SP22J/128/08) was approved by the IRB committee of King Saud bin Abdulaziz University for Health Sciences. The studies were conducted in accordance with the local legislation and institutional requirements. The participants provided their written informed consent to participate in this study.

## Author contributions

RaA: Conceptualization, Investigation, Resources, Supervision, Writing – original draft. SA: Data curation, Writing – original draft. WA: Data curation, Writing – original draft. ReA: Data curation, Writing – original draft. MA: Project administration, Supervision, Validation, Visualization, Writing – review & editing. ZA: Formal Analysis, Investigation, Methodology, Project administration, Software, Supervision, Validation, Visualization, Writing – review & editing.
